# Supplementary material for: A humanized mouse model for adeno-associated viral gene therapy
Source: Nat Commun. 2024 Mar 4;15:1955. doi: 10.1038/s41467-024-46017-0 (PMC10912671; doi:10.1038/s41467-024-46017-0)
Supplement: Supplementary file 1 — Supplementary Information [file 41467_2024_46017_MOESM1_ESM.pdf]

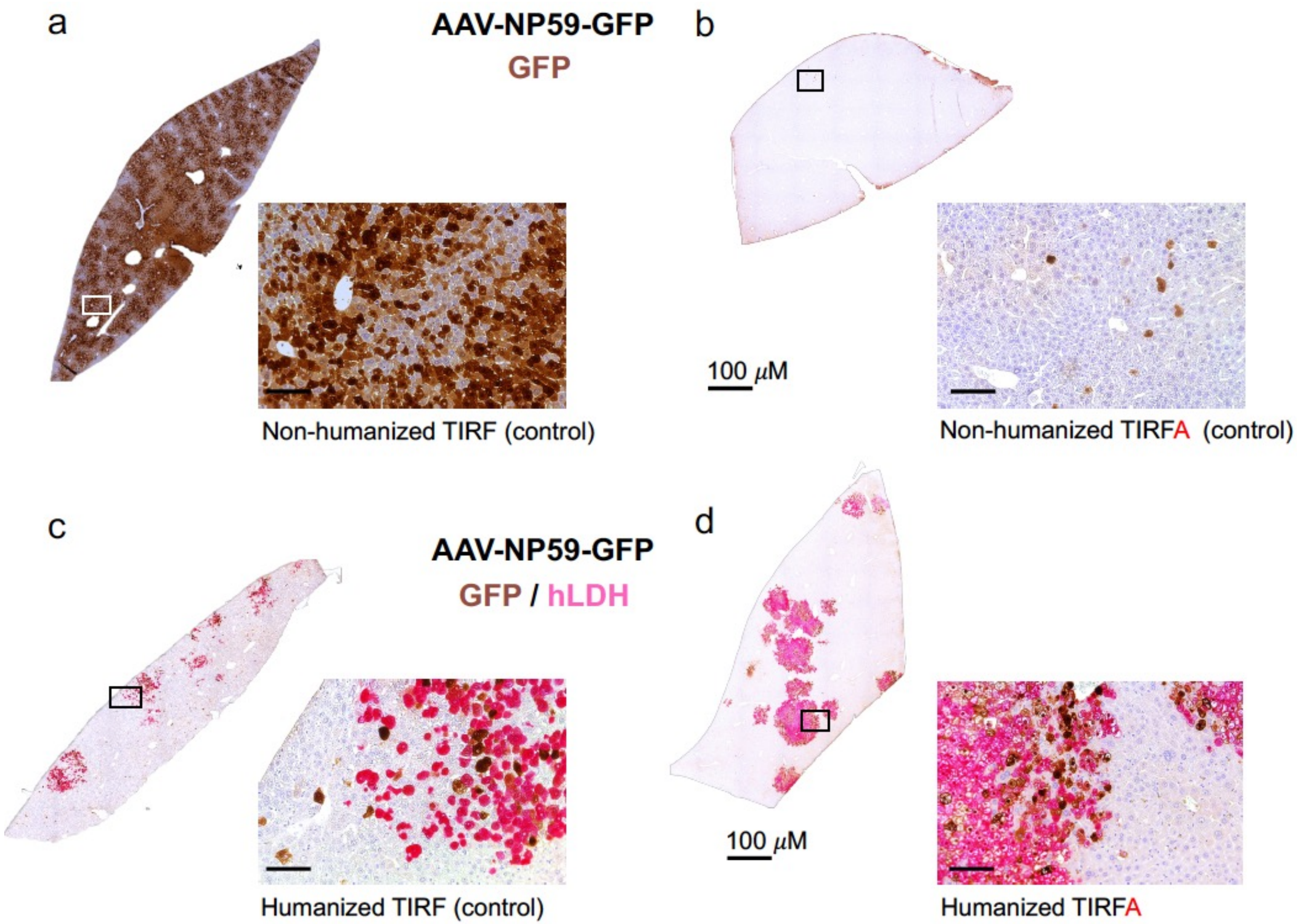

Supplementary Figure 1. Transduction of AAVNP50 in humanized mice. Intravenous injection of $1x10^{12}$ gc/mouse AAVNP59-GFP followed by harvest after one month. Figure shows immunohistochemistry of liver section of co-stained for hLDH (magenta) and the AAV transgene, GFP (brown) in non-humanized TIRF **a** and TIRFA **b** control mice, and humanized TIRF **c** and TIRFA **d** mice. Boxed area show with higher magnification . GFP Green fluorescent protein. hLDL; human lactate dehydrogenase.

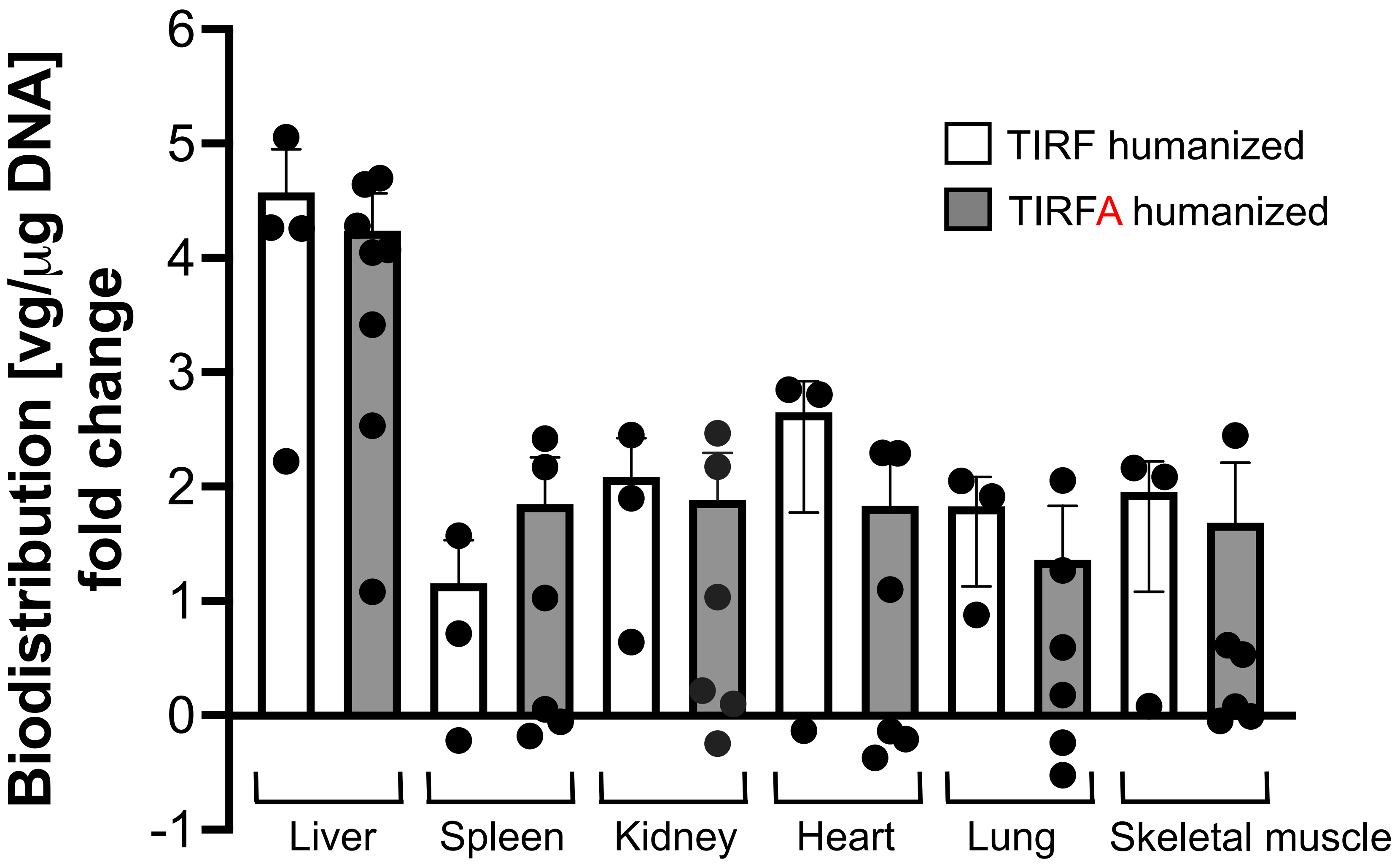

Supplementary Figure 2. Biodistribution of AAV8 viral genome copies in different organs of humanized TIRF and TIRFA mice. Data is expressed in mean ± SEM. Source data are provided as a Source Data file

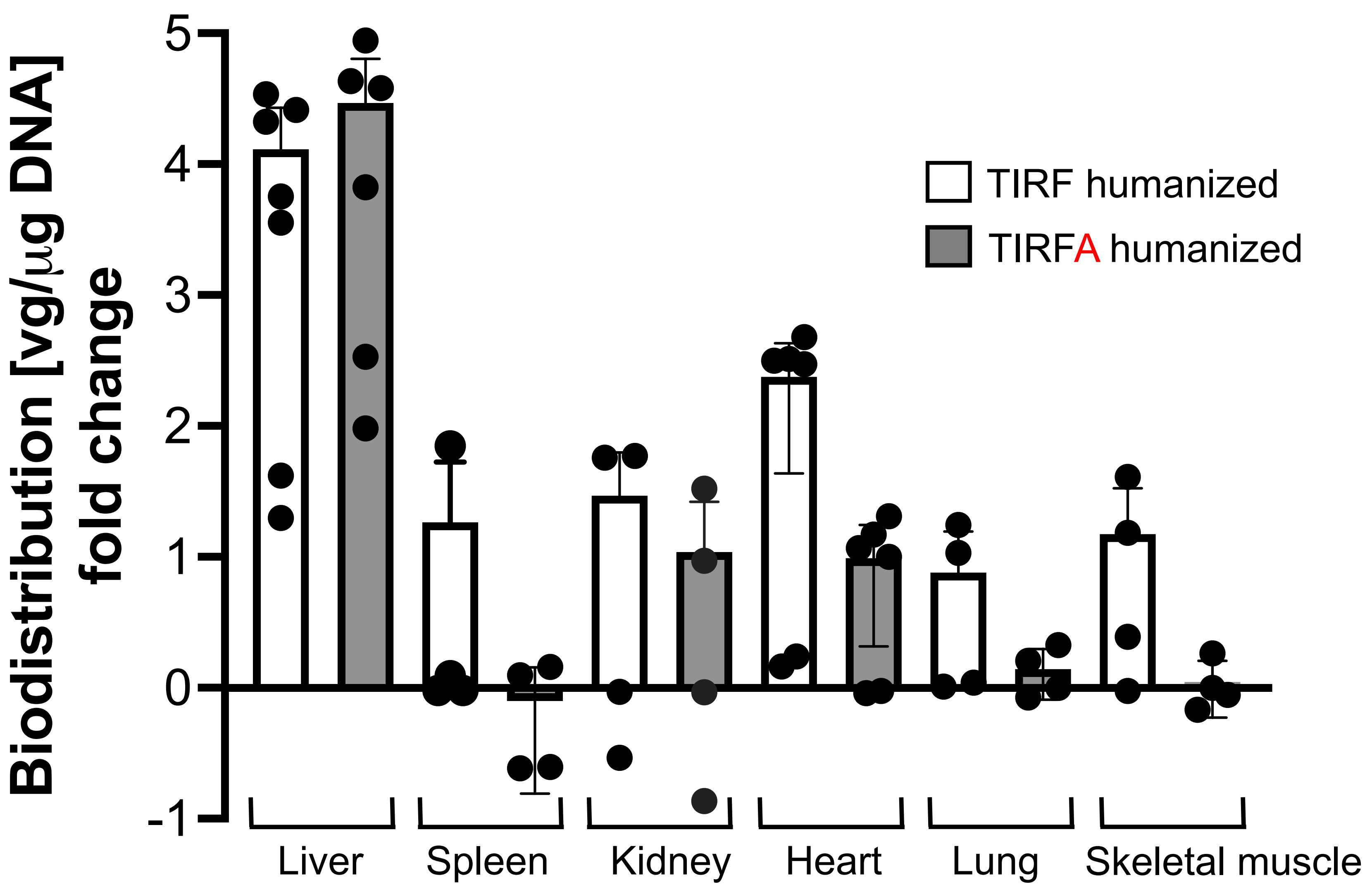

Supplementary Figure 3. Biodistribution of AAV9 viral genome copies in different organs of humanized TIRF and TIRFA mice. Data is expressed in mean ± SEM. Source data are provided as a Source Data file
