# Supplementary material for: A humanized mouse model for adeno-associated viral gene therapy
Source: Nat Commun. 2024 Mar 4;15:1955. doi: 10.1038/s41467-024-46017-0 (PMC10912671; doi:10.1038/s41467-024-46017-0)
Supplement: Supplementary file 3 — Reporting Summary [file 41467_2024_46017_MOESM3_ESM.pdf]

nature portfolio

Corresponding author(s): Karl-Dimiter Bissig

Last updated by author(s): Nov 24, 2023

Reporting Summary

Nature Portfolio wishes to improve the reproducibility of the work that we publish. This form provides structure for consistency and transparency in reporting. For further information on Nature Portfolio policies, see our Editorial Policies and the Editorial Policy Checklist.

Statistics

For all statistical analyses, confirm that the following items are present in the figure legend, table legend, main text, or Methods section.

n/a	Confirmed	
☐	☒	The exact sample size (*n*) for each experimental group/condition, given as a discrete number and unit of measurement
☐	☒	A statement on whether measurements were taken from distinct samples or whether the same sample was measured repeatedly
☐	☒	The statistical test(s) used AND whether they are one- or two-sided<br>*Only common tests should be described solely by name; describe more complex techniques in the Methods section.*
☐	☒	A description of all covariates tested
☐	☒	A description of any assumptions or corrections, such as tests of normality and adjustment for multiple comparisons
☐	☒	A full description of the statistical parameters including central tendency (e.g. means) or other basic estimates (e.g. regression coefficient) AND variation (e.g. standard deviation) or associated estimates of uncertainty (e.g. confidence intervals)
☐	☒	For null hypothesis testing, the test statistic (e.g. *F*, *t*, *r*) with confidence intervals, effect sizes, degrees of freedom and *P* value noted<br>*Give P values as exact values whenever suitable.*
☒	☐	For Bayesian analysis, information on the choice of priors and Markov chain Monte Carlo settings
☒	☐	For hierarchical and complex designs, identification of the appropriate level for tests and full reporting of outcomes
☒	☐	Estimates of effect sizes (e.g. Cohen's *d*, Pearson's *r*), indicating how they were calculated

Our web collection on statistics for biologists contains articles on many of the points above.

Software and code

Policy information about availability of computer code

Data collection — *Provide a description of all coNommercial, open source and custom code used to collect the data in this study, specifying the version used OR*
No software was used for data collection

Data analysis — PRISM version 10.1.0was used for the statistics, ImageJ software was used for cell quantification

For manuscripts utilizing custom algorithms or software that are central to the research but not yet described in published literature, software must be made available to editors and reviewers. We strongly encourage code deposition in a community repository (e.g. GitHub). See the Nature Portfolio guidelines for submitting code & software for further information.

Data

Policy information about availability of data

All manuscripts must include a data availability statement. This statement should provide the following information, where applicable:

- Accession codes, unique identifiers, or web links for publicly available datasets
- A description of any restrictions on data availability
- For clinical datasets or third party data, please ensure that the statement adheres to our policy

All data is available in the manuscript or the supplementary materials. TIRFA mice will be made available through mouse repositories. Source data are provided with this paper.

Research involving human participants, their data, or biological material

Policy information about studies with human participants or human data. See also policy information about sex, gender (identity/presentation), and sexual orientation and race, ethnicity and racism.

Reporting on sex and gender	n/a
Reporting on race, ethnicity, or other socially relevant groupings	n/a
Population characteristics	n/a
Recruitment	n/a
Ethics oversight	n/a

Note that full information on the approval of the study protocol must also be provided in the manuscript.

Field-specific reporting

Please select the one below that is the best fit for your research. If you are not sure, read the appropriate sections before making your selection.

☒ Life sciences ☐ Behavioural & social sciences ☐ Ecological, evolutionary & environmental sciences

For a reference copy of the document with all sections, see nature.com/documents/nr-reporting-summary-flat.pdf

Life sciences study design

All studies must disclose on these points even when the disclosure is negative.

Sample size	no sample size calculations were were done. Inclusion and number of humanized TIRFA mice was based on genotype (homozygous for AAVR-/-) and availability of human hepatocytes (TIRF and TIRFA).
Data exclusions	Except for Figure 1C (human albumin distribution in humanized mice), humanized mice with human albumin levels below 0.1mg/ml in the murine serum were excluded from the study.
Replication	All humanized TIRF and TIRFA mice injected with AAV (AAV8 and AAV9 and NP-59) were validated by immunostaining and generated very similar results (successful replication).
Randomization	Randomization was done based on human albumin levels in the murine blood, e.g. TIRF and TIRFA mice
Blinding	Investigators were not blinded during the whole study with the exception of quantification of immunostaining for AAV8 and AAV9 qPCR as well as mRNA and DNA ISH experiments were also blinded. The rest of experiments didn't require blinding

Reporting for specific materials, systems and methods

We require information from authors about some types of materials, experimental systems and methods used in many studies. Here, indicate whether each material, system or method listed is relevant to your study. If you are not sure if a list item applies to your research, read the appropriate section before selecting a response.

Materials & experimental systems

n/a	Involved in the study	
☐	☒	Antibodies
☒	☐	Eukaryotic cell lines
☒	☐	Palaeontology and archaeology
☐	☒	Animals and other organisms
☒	☐	Clinical data
☒	☐	Dual use research of concern
☒	☐	Plants

Methods

n/a	Involved in the study	
☒	☐	ChIP-seq
☒	☐	Flow cytometry
☒	☐	MRI-based neuroimaging

Antibodies

Antibodies used	Primary antibodies: Mouse anti-LDH (Santa Cruz, sc-133123), Rabbit anti-RFP (Rockland,600-401-379), Chicken anti-GFP (Abcam,ab13970), Rabbit anti-

Antibodies used	Sox17 (Millipore 09-038-1), Rabit anti-Snail (Abcam, ab17732), anti-Rabbit Cytokeratin 14 (Abcam, ab51054), Rabbit anti-Cytokeratin 19 (Abcam, ab52625), Rabbit anti-FAS (Santa Cruz, sc-715), Rabbit anti-GS (Abcam, ab73593). Secondary antibodies: donkey anti-Rabbit Alexa 594 (Thermo Fisher, A21207), donkey anti-mouse Alexa 488 (Jackson ImmunoResearch 715-545-150), donkey anti-chicken Alexa 488 (Jackson ImmunoResearch, 703-545-155). Anti-chicken biotinylated antibody (Vector labs, BA-9010). IHC kit containing horseradish peroxidase and alkaline phosphatase: ImmPRESS duet kit (Vector labs, MP-7714-15).
Validation	Mouse anti-LDH (Santa Cruz, sc-133123, dilution 1:100) was used to detect human cells and human tumors in liver samples of liver humanized mice (TIRF and TIRFA). This antibody was used in combination with rabbit anti-RFP or chicken anti-GFP antibodies and it was developed using IF secondary antibodies (LDH/RFP) and/or IHC double staining kit (Vector Labs ImmPRESS Duet, MP-7714-15).<br><br>Rabbit anti-RFP (Rockland,600-401-379, diltution 1:100) was used to detect td-Tomato in humanized mice (TIRF and TIRFA) injected with AAV8td-Tomato or AAV9-td-Tomato in combination with mouse anti-LDH antibody. The signal was developed by IF and by IHC double staining (Vector labs ImmPRESS Duet, MP-7714-15).<br><br>Chicken anti-GFP (Abcam,ab13970): was used to detect GFP in humanized mice (TIRF or TIRFA) injected with AAVNP59 virus (liver samples) at a dilution of 1:1,500 in combination with mouse anti-LDH antibody and the signal was developed by IHC double staining (anti-chicken biotinylated secondary antibody for GFP and Vector labs ImmPRESS Duet, MP-7714-15 for LDH). This antibody was also used to detect GFP in teratoma samples of mice injected with AAV9-GFP virus at a concentration of 1:200 and in combination with different germ-layer/differentiation markers and the signal was developed by IF using anti-chicken Alexa-488 secondary antibody).<br><br>Rabbit anti-Sox17 (Millipore 09-038-1) diluted 1:200, Rabbit anti-Snail (Abcam, ab17732) diluted 1:1000, anti-Rabbit Cytokeratin 14 (Abcam, ab51054) diluted 1:1000, Rabbit anti-Cytokeratin 19 (Abcam, ab52625) diluted 1:1000, Rabbit anti-FAS (Santa Cruz, sc-715) diluted 1:250, Rabbit anti-GS (Abcam, ab73593) diluted 1:1000: all these antibodies were used in combination with chicken anti-GFP antibody (diluted 1:500) to detect GFP expression in different areas of the teratoma in mice injected with AAV9-GFP. The signal was developed by IF (GFP in green channel and the other markers in red) using the corresponding secondary antibodies diluted 1:1000.

Animals and other research organisms

Policy information about studies involving animals; ARRIVE guidelines recommended for reporting animal research, and Sex and Gender in Research

Laboratory animals	We used 8-week old TIRF (Il2rg-/-/Rag2-/-/Fah-/-) and TIRFA Il2rg-/-/Rag2-/-/Fah-/-/Aavr-/-) mouse strains. All aminals were maintained under a standard 12-h dark/light cycle with water and chow (Picolab Rodent diet 20, LabDiet, cat# 5053) provided ad libitum.
Wild animals	No wild animals were used in this study
Reporting on sex	N/A
Field-collected samples	No field collected samples were used in the study.
Ethics oversight	All animal experiments were approved by the Duke University Institutional Animal Care and Use Committee

Note that full information on the approval of the study protocol must also be provided in the manuscript.

Plants

Seed stocks	N/A *Report on the source of all seed stocks or other plant material used. If applicable, state the seed stock centre and catalogue number. If plant specimens were collected from the field, describe the collection location, date and sampling procedures.*
Novel plant genotypes	N/A *Describe the methods by which all novel plant genotypes were produced. This includes those generated by transgenic approaches, gene editing, chemical/radiation-based mutagenesis and hybridization. For transgenic lines, describe the transformation method, the number of independent lines analyzed and the generation upon which experiments were performed. For gene-edited lines, describe the editor used, the endogenous sequence targeted for editing, the targeting guide RNA sequence (if applicable) and how the editor was applied.*
Authentication	N/A *Describe any authentication procedures for each seed stock used or novel genotype generated. Describe any experiments used to assess the effect of a mutation and, where applicable, how potential secondary effects (e.g. second site T-DNA insertions, mosiacism, off-target gene editing) were examined.*
